# Prevalence of cardio-respiratory factors in the occurrence of the decrease in oxygen uptake during supra-maximal, constant-power exercise

**DOI:** 10.1186/2193-1801-2-651

**Published:** 2013-12-05

**Authors:** Christine Hanon, Sylvain Dorel, Rémi Delfour-Peyrethon, Pierre-Marie Leprêtre, David J Bishop, Stéphane Perrey, Claire Thomas

**Affiliations:** French National Institute of Sports (INSEP), Research Department, Laboratory of Sport, Expertise and Performance, 11 Tremblay Avenue, 75012 Paris, France; Laboratory “Motricité, Interactions, Performance” (EA 4334), University of Nantes, F-44000 Nantes, France; University of Picardie Jules Verne (EA-3300), 80025 Amiens cedex, France; Movement to Health (M2H), Montpellier-1 University, Euromov, Montpellier, France; Institute of Sport, Exercise and Active Living (ISEAL), and the College of Sport and Exercise Science, Victoria University, Melbourne, Australia; STAPS Department, University of Evry Val d’Essonne, François Mitterrand Boulevard, 91025 Evry, France

**Keywords:** High-intensity exercise, Oxygen consumption, Acidosis, Cardio-respiratory parameters

## Abstract

**Purpose:**

To investigate the physiological mechanisms that explain the end-exercise decrease in oxygen uptake  during strenuous constant-power exercise, we recruited eleven trained, track cyclists.

**Methods:**

On two separated days they performed 1) resting spirometric measures, followed by an incremental test on a cycle ergometer to determine the power output at  and 2) an exhaustive isokinetic supramaximal cycling exercise (Tlim_supra_) at 185 ± 24% of  (i.e., 640.5 ± 50.8 W). During cycling exercise tests, , ventilation parameters, stroke volume (SV) and heart rate were continuously recorded. Furthermore, arterialised capillary blood samples were collected to measure blood pH, arterial oxygen saturation, lactate and bicarbonate concentration before and 5 min after Tlim_supra_.

**Results:**

A > 5% decrease in  and/or SV was observed in 6 subjects, with 5 out of 6 subjects presenting both phenomena. The magnitude of the  decrease was correlated with the magnitude of the SV decrease (R = 0.75, P < 0.01), the peak-exercise end-tidal O_2_ partial pressure (R = 0.80, P < 0.005) and the resting, forced expiratory volume in 1 s (R = 0.72, P < 0.05), but not with any blood variables. The significant post-Tlim_supra_ decrease in forced vital capacity and forced inspiratory volume corroborate with a possible respiratory muscle fatigue.

**Conclusion:**

Based on these findings, we demonstrate that the occurrence of  decrease in more than half of our subjects, during a strenuous constant-power exercise leading to a mild-acidosis (pH = 7.21 ± 0.04), results mainly from cardio-respiratory factors and not from blood metabolic responses.

## Background

A significant decrease in whole-body pulmonary oxygen uptake  at the end of supra-maximal running exercise in the field has been reported (Billat et al. 2009 Hanon et al. 2010; Thomas et al. 2005). Of note, the  decrease was concomitant with a decrease in running velocity that could logically be considered as one of the explanations for this phenomenon. However, it is important to note that (i) the  decrease was proportionally larger than the drop in running velocity (Hanon and Thomas 2011) and (ii) the final velocity was always greater than the velocity associated with the maximal  of each subject (Hanon and Thomas 2011). Additionally, researchers have also observed a  decrease during exhaustive treadmill exercise performed at a constant intensity (Nummela and Rusko 1995), (Perrey et al. 2002). An important unresolved physiological question therefore, is what are the mechanisms that contribute to this phenomenon?

Gathering their 400-, 800- and 1500-m data, Hanon et al. (Hanon and Thomas 2011), established correlations between the peak blood lactate concentration ([La]) (R = 0.55, P < 0.05) and the magnitude of the  decrease, and between the 300-m intermediate pH value and the 400-m final  (R = 0.86) (Hanon et al. 2010). Low blood pH values reduce the affinity of O_2_ to haemoglobin and contribute to an exercise-induced arterial hypoxemia (EIAH). Harms et al. (Harms et al. 2000) stated that  appears to decrease by 2% for each 1% decrease of arterial O_2_ saturation (SaO_2_), at least when SaO_2_ is less than 95%. Furthermore, acid–base disturbances have been shown to change the partial pressure at which carbon dioxide begins to stimulate breathing (Duffin 2005). The model simulations presented by this author (Duffin 2005) demonstrated the importance of the central strong ions difference (SID) in the regulation of breathing. Therefore, an altered acid–base balance in response to supra-maximal exercise may also contribute to disturbances in exercise ventilation, O_2_ transport, and utilisation. Independant of changes in blood pH, increases in blood lactate levels have also been associated with decreases in oxygen supply (Rozier et al. 2007 Mortensen et al. 2007) and O_2_ extraction (Poole et al. 1994). Therefore, large ionic and metabolic perturbations at the end of exhaustive supra-maximal exercise may affect O_2_ transport and utilisation and contribute to the end-exercise  decrease.

Concomitant with the decrease in  observed during exercise performed at ~95% of , Perey et al. (Perrey et al. 2002) observed a decrease in minute ventilation , tidal volume (V_T_). Hanon & Thomas (Hanon and Thomas 2011) also reported a strong correlation between the V_T_ and  responses observed in the last 100 m of 400-, 800-, and 1500-m races (r = 0.85, P < 0.0001), suggesting that respiratory response patterns may play a role in the  decrease during the latter part of supra-maximal exercise. With little increase in alveolar O_2_ pressure (PAO_2_) during exercise, the pulmonary diffusion capacity becomes critical for the maintenance of arterial O_2_ pressure (PaO_2_) (Dempsey 2006), and large lungs appear to be an advantage when performing whole-body exercise (Nielsen 2003). On the other hand, large swings in thorax movement could present negative consequences such as excessive fluctuations in intra-thoracic pressures (Amann 2011) or extreme respiratory muscle work and fatigue (Aaron et al. 1992). Indeed, many studies having shown that the respiratory system might affect the quality of the O_2_ transport during strenuous exercises (Nielsen 2003), it remains to test the contribution of end-exercise respiratory response on the  decrease.

The significant  decline observed in the last two minutes of a 5–10 min exhaustive test (Gonzalez-Alonso and Calbet 2003) has also been directly associated with the inability of the heart to maintain the rate of O_2_ delivery to locomotive skeletal muscles. These authors emphasised that the mechanisms of fatigue which could explain the declining systemic O_2_ delivery and  during heavy exercise were complex, possibly involving inhibitory signals that originated in different bodily tissues and organs. However, these authors did not concurrently measure changes in respiratory variables, and, further, it is not known if changes in cardiac parameters also contribute to the exercise-induced decrease in  during supra-maximal exercise lasting less than 2 min. The link between resting lung volumes, exercise-induced cardio-respiratory responses on one hand, and the decrease in  on the other, needs to be investigated.

Therefore, the main aims of this study were to identify the primary factors associated with the inability to maintain a high steady-state  in healthy, trained subjects. To rule out the potential confounding influence of a decrease in velocity or power output, we chose a constant-work-load cycle exercise. The subjects were tested on a cycle ergometer in order to control the pedalling pattern as participants fatigued (i.e., to avoid a frequency and then a power decrease). We hypothesized that the impairment of both cardiac and respiratory function associated to metabolic perturbations would result in a  decrease.

## Results

The results are expressed as the group average, with corresponding statistical results, and for the main physiological variables, as the individual responses referenced as a letter (A to K).

### Torque velocity and incremental tests

Mean values for P_max_ and *f*_opt_ were 1,318 ± 191 W and 121 ± 7 rpm, respectively.  and  corresponded to 4.2 ± 0.7 L.min^-1^ (57.9 ± 6.9 mL.min^-1^.kg^-1^) and 350 ± 32 W, respectively. The maximal blood lactate value measured at the end of the incremental test was 13.1 ± 2.5 mmol.L^-1^. Maximal CO, SV and HR were 25.1 ± 1.5 L.min^-1^, 132.2 ± 13.2 mL.beat^-1^ and 188 ± 10 beats.min^-1^, respectively.

### Tlim_supra_ test

The mean performance for T-lim_supra_ test was 51.4 ± 6.9 s (range from 43 to 65 s). During this test, a mean power (P_supra_Δ30%) of 641 ± 51 W was sustained at a mean pedalling rate of 109 ± 6 rpm; the mean power output corresponded to 185 ± 24% of  and 49 ± 3.8% of P_max_.

### Respiratory responses

**Figure 1:**
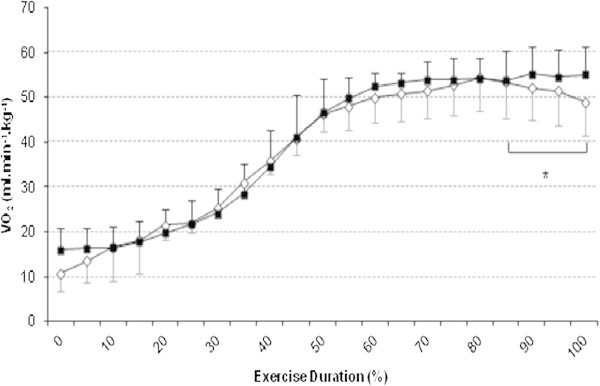
**Mean time course of**  **during the Tlim**_**supra**_ **test in the decrease and no decrease subjects.** Time course of oxygen uptake during the Tlim_supra_ test at each 5% interval-duration in the subjects who present a ≤ 5% (white labels) and > (black labels)  decrease Values are mean ± SD; * : significant decrease relative to , *P* < 0.05.

The  reached during the Tlim_supra_ test was equal to 55.0 ± 7.3 mL.min^-1^.kg^-1^ (95.0 ± 7.6% of ). Figure 1 displays the time course of the  expressed relative to time for the eleven subjects. During Tlim_supra_, a  value was detected at 43.3 ± 5.3 s after the onset of the test (~80% of the total test duration). From 80% of the total duration until the end of the test, average mean  for the group, significantly decreased by 5.4 ± 4.7% of the  (*P* < 0.05). The peak VRMO_2_ value observed at the end of the exercise was 423.9 ± 96.7 mL.min^-1^. This corresponded to 11.9 ± 2.1% (ranged from 8.6 to 15.0%) of the whole pulmonary oxygen uptake.

**Figure 2:**
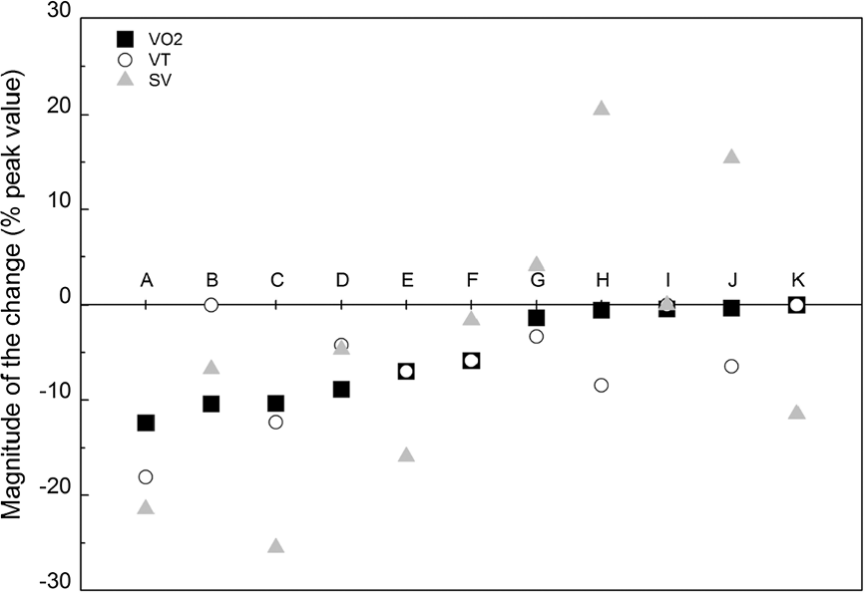
**Magnitude of the**  **, V**_**T**_ **, SV changes in the eleven subjects during the Tlim**_**supra**_ **test.** , V_T_, SV are changes (decrease or increase) expressed relatively to peak values observed during the constant-power cycling test (Y axis in %). **A, B…..K** represent the eleven subjects classified from the greatest (on the left) to the smallest (on the right)  decrease. Six subjects (A to F) presented a > 5% decrease in  and/or SV.

In 6 of our 11 subjects (subjects A, B, C, D, E, F in Figure 2), the decrease in  was greater than 5%, corresponding to 9.1 ± 2.4% of peak values. In the 5 other subjects (G, H, I, J, K), the decrease was between zero and 3.5% (0.9 ± 2.0%).

During the Tlim_supra_, considering the peak (2.6 ± 0.5 L) and final values (2.4 ± 0.4 L), a global decrease in V_T_ corresponding to 5.9 ± 5.6% was found (*P* < 0.05) with no concomitant global decrease in RF and . This V_T_ decrease was observed in 7 subjects (Figure 2), whereas a decrease in RF and in  (not presented in Figure 2) was only observed in one (subject E) and 3 subjects (subjects A, E and J), respectively. The decrease in VT was 7.9 ± 6.4% in subjects who present a  decrease (A to F) and 3.5 ± 3.8% in subjects G to K who present a <5%  decrease. The difference between these two groups was significant (*P* < 0.05) with an effect size equivalent to 0.80.

The functional pulmonary data are presented in Table 1. The ICC for the FVC pre-tests was 0.95 (confidence interval: 0.88-0.98). The mean P_ET_O_2_ peak value was 122.2 ± 4.8 mmHg and VR was 89.8 ± 10.5% of the estimated MVV. The difference between the subjects who exhibited a < or > 5%  decrease was significant (*P* < 0.05) for the pre-exercise values of FEV_1_ with a corresponding effect size equivalent to 2.24.

**Table 1:** **Mean (SD) spirometric data measured at rest (pre-test) and 3 min after exercise (post-test)**

	Pre-test	Post-test	Post-test (%)
FVC	5.2 (0.8)	5.0 (0.8) *	96.4 (6.0)
FEV_1_ (L)	4.4 (0.6)	4.2 (0.7) †	95.5 (7.0)
FEV_1_/FVC (%)	85.7 (7.6)	84.7 (8.9)	99.2 (9.7)
FIVC (L)	5.6 (0.7)	5.4 (0.9) *	96.6 (4.7)
FIV_1_ (L)	5.3 (0.6)	4.7 (1.3)	90.7 (21.0)
FEV _25_ (L.s^-1^)	6.6 (1.5)	5.2 (1.9) *	86.2 (32.6)
FEV _50_ (L.s^-1^)	4.8 (0.8)	4.7 (1.2)	98.5 (19.5)
FEV _75_ (L.s^-1^)	2.6 (0.9)	3.0 (1.2)	108.5 (32.3)

*: Significant difference between pre and post test at P < 0.05, †: P = 0.08.

*Post-test (%)* post-test expressed in % pre-test, *FVC* forced vital capacity, *FEV*_*1*_ forced expiratory volume in 1 s, *FEV*_*1*_ */FVC, FIVC* forced inspiratory volume, *FIV*_*1*_ forced inspiratory volume in 1 s, *FEV*_*25, 50, 75*_ forced expiratory flow at that point that is 25, 50 and 75% from FVC.

**Figure 3:**
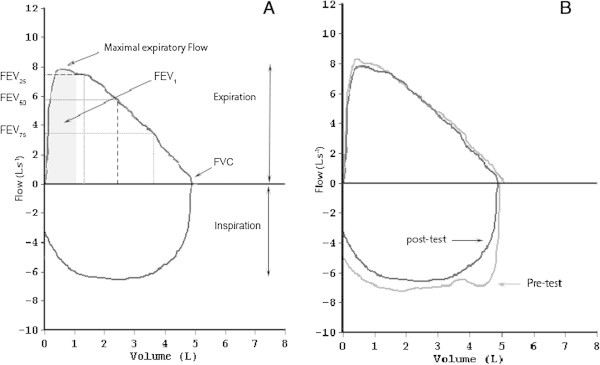
**A. Main spirometric parameters recorded during the experimental session, B. Maximal flow-volume loop measured at rest before the Tlim**_**supra**_ **and 3 min after the Tlim**_**supra**_ **in subject D. A**: FVC: forced vital capacity, FEV_1_: forced expiratory volume in 1 s, FEV_25, 50, 75_: forced expiratory flow at that point that is 25, 50 and 75% from FVC. **B**: in grey and black: maximal flow volume recorded before and after the Tlim_supra_ test, respectively.

The comparison between pre and post-Tlim_supra_ (Figure 3) data revealed a significant decrease in FEV_25_, FIVC and FVC (*P* < 0.05).

#### Cardiac responses

HR values attained a steady-state value of 185 ± 11 beats.min^-1^ (98.4 ± 5.0% of IT maximal HR). The highest CO (25.0 ± 5.6 L.min^-1^) and SV (140.3 ± 33.0 mL) mean values measured during Tlim_supra_ were not different from maximal values recorded during the IT. In 6 out of 11 subjects (A, B, C, D, E and K), a SV decrease of more than 5% was observed (Figure 2).

If we compare the decrease in SV in those subjects who present a  decrease or not, the decrease values in SV were 17.7 ± 12.3% in subjects A to F and 3.8 ± 8.4% in subjects G to K. The difference between the two groups was significant (*P* < 0.05) with a corresponding effect size equivalent to 1.29.

#### Blood metabolic responses

The blood results measured before and after Tlim_supra_ are presented in Table 2. The peak values of [La], pH and [HCO_3_^-^] were obtained 5 min after the end of the exercise. The SaO_2_ value measured immediately after stopping exercise was 92.5 ± 2.7%.

**Table 2:** **Mean (SD) values for blood parameters measured during the Tlim**_**supra**_ **test**

	Pre-test	0	5 min	8 min
SaO_2_ (%)	95.2 (1.5)	92.5 (2.7) *	95.9 (1.0) *	95.7 (1.2)
[La] (mmol.L^-1^)	3.8 (1.1)	9.4 (3.3) *	15.9 (1.7) *	14.9 (1.9) *
pH	7.39 (0.02)	7.30 (0.06) *	7.21 (0.04)*	7.24 (0.04)*
[HCO_3_^-^] (mmol.L^-1^)	23.7 (1.1)	20.7 (2.5) *	12.3 (1.8) *	12.4 (1.8)
paO_2_ (mmHg)	78.9 (9.2)	76.2 (15.5) *	99.0 (8.5) *	95.9 (14.0)
paCO_2_ (mmHg)	38.8 (1.6)	42.0 (3.1) *	30.7 (2.9) *	28.9 (2.8) *

* statistically different from the previous result (post 5 is different from post 0,…, post 8 from post 5). P < 0.05.

The blood parameters were collected 1 min before the test at the end of the warm-up (pre-test), and at 0, 5 and 8 min of passive recovery following the Tlim_supra_ test.

The blood variables are oxygen saturation (SaO_2_), lactate concentration [La] and bicarbonate concentration [HCO_3_^-^], pH and partial pressure for O_2_ (PaO_2_) and CO_2_ (PaCO_2_). n = 11.

#### *Relationships between the*decrease and metabolic, respiratory and cardio-dynamic data

**Figure 4:**
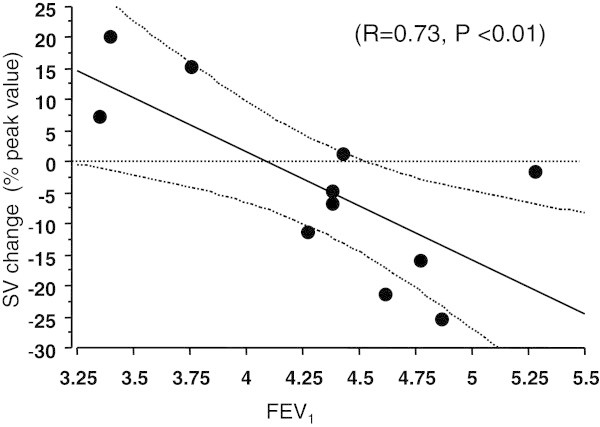
**Relationship between the magnitude of the decrease in stroke volume (SV) and the resting forced expiratory volume in 1 s (FEV**_**1**_ **).** FEV_1_ (L): forced expiratory volume in 1 s, SV change (%): stroke volume expressed as a percent of peak value (r = 0.73, P < 0.01), n = 11. The broken lines indicate the 95% confidence intervals.

The magnitude of the  decrease was correlated with the P_ET_O_2_ peak values (R = 0.80, P < 0.005), and the correlation with the decrease in V_T_ approached significance (R = 0.57, P = 0.06). The magnitude of the  decrease was also correlated with FEV_1_ (R = 0.72, P < 0.005) and FEV_25_ (R = 0.73, P < 0.01) measured at rest and post-exercise, respectively. The partial correlations between  on one part and V_T_, SV, P_ET_O_2_ and FEV_1_ on the other part, were 0.52, (P > 0.05), 0.70, 0.78 and 0.71 (P < 0.05), respectively. As observed in Figure 2, 5 of the 6 subjects exhibiting a  decrease also presented a SV decrease (expressed as a percentage of the peak value) (8.6 ± 9.9 mL.beat^-1^), but the inverse was not verified with one subject (K) presenting a drop in SV without a  decrease. Nevertheless, the relationship between the SV and  decrease was significant (R = 0.75, P < 0.01). Significant correlations were also observed between SV decrease and both the peak value of P_ET_O_2_ (R = -0.65, P < 0.05) and the resting FEV_1_ (R = 0.73, P < 0.01) as shown in Figure 4. No significant relationships (*P* > 0.05) were observed between the  decrease and the blood data ([La] (R = -0.45), pH (R = 0.10), SaO_2_ (R = 0.14) and [HCO_3_^-^] (R = 0.24).

## Discussion

A significant mean decrease in  was observed in the last 20% of the total exercise duration. This decrease was greater than 5% of the peak value in 6 out of 11 subjects, with 5 of these 6 subjects also presenting a decrease in stroke volume. The correlations indicated that the magnitude of the  decrease was linked with that of SV, and that both were negatively linked with respiratory parameters such as peak exercise end tidal O_2_ partial pressure and resting forced expiratory volume in 1 s. The strong interrelations between cardiac and respiratory responses suggest that both contribute to the  decrease during intense, supra-maximal cycling exercise. A significant post-exercise decrease in resting expiratory and inspiratory flow volumes was observed suggesting that there was also respiratory muscle fatigue.

### VO_2_ peak

The present study indicates that during a cycling test performed at 185% of MAP, well-trained cyclists are able to reach 95% of their  in less than 50 s. This is similar to the value of 94% obtained during a 400-m track run (Hanon et al. 2010). As reviewed by Gastin (Gastin 2001),  can be as high as 90% of the athlete’s maximum after 30–60 s. However, these previous studies all utilized intensive cycling exercise of short duration and initiated with a maximal starting power (Wingate test or all-out exercise). In the present study, the power was constant, but sufficiently elevated (185% ) to induce exhaustion in less than 60 s. Therefore, our protocol was successful at soliciting a large percentage of the  during an intense constant-power exercise in well-trained sprint cyclists.

###  decrease

A moderate decrease in the mean  was observed during the final 20% of the supramaximal cycle test. The magnitude of this  decrease (0 to 12%) differed from our recent results obtained during a 400-m running field test of similar duration (50 s), in which a systematic and greater  drop (15%) was observed in the final 100 m (Hanon et al. 2010). Of note, and contrary to the present study, this last exercise segment was performed with a large velocity decrease. Nevertheless, a  decrease can occur in exercise performed at a constant pace in a subset of subjects, suggesting that at least some of this decrease is independent from a velocity or power decrease (Nummela and Rusko 1995 Perrey et al. 2002). It should be noted that, as in the above-mentioned studies (Hanon et al. 2010), the  decrease occurred while  was not reached.

Each step in the O_2_ supply chain, from breathing air to transport to the muscle cells, could influence O_2_ availability, especially during whole-body, maximal-intensity exercise. Although hyperventilation produces an increase in alveolar O_2_ tension to overcome the diffusion limitation of the lungs (Dempsey 2006), this could also have negative consequences such as extreme energetic cost, respiratory muscle fatigue, or attainment of the respiratory reserve. Each of these factors could have influenced  during the latter stages of our exercise protocol.

### Metabolic data and the  decrease

The lack of a relationship between the magnitude of the  decrease and the post-test blood changes is not in accordance with our previous all-out running data. In this previous experiment, a 23 and 12% drop in velocity was observed in the last 100 m of 400- (Hanon et al. 2010) and 800-m (Hanon and Thomas 2011) races, respectively. The [lactate], [HCO_3_^-^] and pH were respectively 22.0 mmol.L^-1^, <5 mmol.L^-1^ and 7.00 after the 400-m race, whereas these values were 15.9 mmol.L^-1^, >12 mmol.L^-1^ and 7.21 in the present constant-power exercise, indicating a more moderate alteration of the acid–base balance. Therefore, in this context, we can hypothesize that the blood buffers were not completely depleted with the result that, contrary to the running (Hanon et al. 2010), rowing (Nielsen et al. 1999) or cycling (Bishop et al. 2007) all-out exercises, the organism was able to prevent an additional acidosis. In the present study, the post-exercise arterial saturation values (92.5 ± 2.7%) are at the limit of the definition of EIAH (less or equal to 92%). The magnitude of the  decrease (5.4%) appears to be in line with the statement that  appears to decrease by 2% for each 1% decrease of SaO_2_ under 95% (Harms et al. 2000). Nevertheless, no significant correlation was observed between the magnitude of the  decrease and the present blood PaO_2_, SaO_2_ and pH values. The brief duration of this supra-maximal exercise, the type of exercise (constant-power vs all-out), and the chosen sport (cycling vs. running), could explain the lower EIAH values compared to those usually observed in well-trained runners (Millet et al. 2009). These global metabolic results suggest that if the bicarbonate reserve are sufficient to eliminate excess H^+^, the O_2_ saturation may not be maximally affected by the eventual decrease in PaO_2_ (Nielsen 2003) and may not represent a major cause of the decrease in .

### Respiratory cost and respiratory muscle fatigue

During a 10-min exercise at ~95% of , Perrey et al. (Perrey et al. 2002) observed a significant decrease in  (due to a decrease in V_T_) in subjects who demonstrated a  decrease. In the present supra-maximal exercise,  and RF increased until the end of the exercise, except in two subjects who exhibited a concomitant  and  decrease. However, the overall significant V_T_ decrease (5%), observed in eight subjects at the end of the test, tended to be correlated with the decrease in  (R = 0.57, P = 0.06, n = 11). The maximal VRMO_2_ values (9–15% of the whole pulmonary ), similar to the maximal values previously published (Aaron et al. 1992) and the VR values (90 ± 10% of MVV), could also raise questions about the ability to carry out this ventilatory load. Furthermore, the functional capacity tests demonstrated a decrease in the inspiratory forced capacity after the Tlim_supra_. This result is in line with that recorded in well-trained rowers (Volianitis et al. 2001), cyclists (Romer et al. 2006) and swimmers (Lomax and McConnell 2003) who experienced a reduction in inspiratory muscle strength immediately after exercise. The magnitude of this decrease in the present study (10%) was less than post 300- and 400-m swimming (15%), but this latter measurement was performed 20 s after the end of the test. Based on the observation that voluntary activation recovers almost fully by 3 min (Bigland-Ritchie et al. 1986), we chose to collect post-test spirometric data 3 min after the exercise in order to exclude the hypothesis of a central activation failure. Our data demonstrating a FIVC decrease are in line with the observation of the diaphragm fatigue shown by Johnson et al. (Johnson and Sieck 1993) who stated that near maximal VR values cannot be carried out for more than 15 to 30 s. Therefore, our data confirm that the respiratory muscle response is likely to be affected during constant-power supra-maximal exercise.

### Respiratory reserve

Maintaining the O_2_ alveolar pressure (P_A_O_2_) through the stimulation of the respiratory muscles could cause athletes to reach and even surpass the respiratory reserve during maximal exercise, and a small portion of the maximal exercise flow volume and pressure-volume envelope on expiration could approach maximal expiratory flow limits near end-expiratory lung volume (Johnson et al. 1996). In the present study, only one subject reached the resting VR values and this subject did not exhibit a  decrease but, Babb (Babb 2013) stated that expiratory flow limitation is not all or none phenomenon and that approaching maximal expiratory flow can affect breathing mechanics. The onset of dynamic airways compression and subsequent airway resistance start long before expiratory flow becomes limited. Therefore in the last part of the exercise, when near  values are attained, a number of mechanisms for inadequate hyperventilation are possible (Johnson et al. 1992). Furthermore, based on the demonstration of a modified  response in an inclined versus an upright position (Grappe et al. 1998), we cannot exclude an influence of the inclined cycling position on the ratio between  recorded in the cycling position and the MVV recorded in an upright position.

### Cardio-respiratory responses and  decrease

All subjects who exhibited a decrease in  also presented a decrease in SV during the exercise, and a correlation was observed between the final SV data and the decrease in . The observation that CO declined significantly before maximal heart rate was reached confirms the results presented by Gonzales-Alonso (Gonzalez-Alonso and Calbet 2003) and indicates that maximal cardiovascular function was attained below maximal heart rate. The decline in stroke volume clearly caused the drop in CO, although the underlying mechanisms remain obscure. The positive correlation between the decrease in  and FEV_1_ could indicate that expiratory intrathoracic pressure could have a negative effect on the  response. Because the heart and lungs share a common surface area, progressive lung inflation and hyperpnea with exercise may increase competition for intrathoracic space and inhibit cardiac filling via a change in cardiac compliance (Peters et al. 1989). Expiratory load leads to a reduction in CO related to an increase in expiratory abdominal and intrathoracic pressure (Stark-Leyva et al. 2004). Hortop et al. (Hortop et al. 1988) has previously demonstrated, in patients with a cystic fibrosis, a strong relationship between the changes in SV with exercise and the FEV_1_. In our trained subjects, the decrease in SV was significantly correlated with P_ET_O_2_ and FEV_1_, which could corroborate the relationship reported between SV and changes in intrathoracic pressure following voluntary lung inflation (Stark-Leyva et al. 2004) and the findings of a recent overview emphasizing the respiratory mechanisms that impair O_2_ transport (Amann 2011). In those subjects with high levels of expiratory flow, we could suggest that, in inclined cycling position, positive expiratory intrathoracic pressure is greater, increasing the ventricular afterload and reducing the rate of ventricular filling during diastole (Miller et al. 2007 Stark-Leyva et al. 2004) which could be deleterious for the maintenance of SV (Amann 2012) and therefore .

## Conclusions

We demonstrated that a  decrease occurs at the end of a constant-power supra-maximal exercise in 6/11 subjects, with the main result being that this phenomenon was related to respiratory characteristics and to the cardiac response. The relationship between stroke volume and  decrease confirms, for supramaximal exercise, previous observations for longer and less intensive cycling exercise (Gonzalez-Alonso and Calbet 2003), (Mortensen et al. 2008). Furthermore, the influence of the respiratory system on the  response observed during the exercise in the participants who present both high resting forced expiratory volume and exercise peak P_ET_O_2_ are innovative and confirm that the pulmonary system is a key determinant of the physiologic responses before stopping a supramaximal cycling exercise. The present data suggest that the respiratory response in case of acute maximal exercise could be the origin of the decrease in SV and  in cycling position. The relation between respiratory, cardiac parameters and  decrease in the case of acute acidosis remains to be tested, and we can hypothesize that different mechanisms may be involved in the  decrease depending on the level of acidosis and the body position.

## Methods

Fourteen specifically trained subjects were solicited for this study. They had at least 5 years of competitive cycling experience and trained 8 hours per week in sprint track cycling and/or BMX. All were successful at national-level events and none had any history of pathology of the lower-limb muscles or joints.

Three subjects were not retained in data processing because of signal loss in the collection of ventilatory data or non observance of the given pedalling rate. Then, eleven trained men (age 24.9 ± 6.5 y, height 1.79 ± 0.05 m and body mass 75.3 ± 8.2 kg) volunteered for this study. They were informed of the nature of the study, and the possible risks and discomforts associated with the experimental procedures, before giving their written consent to participate. The experimental design of the study was approved by the local Ethics Committee of Saint-Germain-en-Laye (France; acceptance n°2009-A01004-53), and was carried out in accordance with the Declaration of Helsinki.

### Experimental protocol

The protocol, carried out during the pre-competition period, included two sessions separated by two days: (1) a first session consisting of anthropometric measurements, resting spirometric monitoring (volume and flow), a torque-velocity cycling test, and an incremental test performed until exhaustion on a calibrated cycle ergometer, (2) a second session consisting of a constant-load, supra-maximal cycle test performed until exhaustion; in a pilot study, we observed that the body temperature was not increased by more than 1°C during this test.

During the first visit, anthropometric data were recorded, subjects were familiarized with the spirometric tests to be performed in this study, and three resting spirometric tests were recorded in order to test the reliability of the measures (Figure 3). Subjects began with a warm-up of 15 min of cycling at 100–150 W, 1 min of recovery and a 5-s sprint. After a 5-min recovery, participants were asked to perform three maximal cycling sprints (5 s separated by 3 min of recovery) according to a previous protocol (Dorel et al. 2010). Three different resistive torques of 0, 0.4-0.7, 1–1.5 Nm/kg body mass were applied to obtain maximal force and power values over a large range of pedaling rates among the three bouts. After computation, the data from the three sprints were used to draw force- and power-velocity relationships and hence to determine maximum power (P_max_) and the corresponding specific optimal pedaling rate (*f*_opt_) at which P_max_ occurred (for details, see (Dorel et al. 2010)).

After 20 min of rest, they performed an incremental cycle test (IT) to determine their  and power output at  (, i.e. the power that elicited ). The progressive protocol consisted of 6 min of pedaling at 100 W followed by a stepped ramp increase in power output of 20 W.min^-1^ until volitional exhaustion. Participants were instructed to maintain their chosen preferred cadence for as long as possible, and the test was completed when the cadence fell more than 10 rpm below this value for more than 5 s despite strong verbal encouragement. All respiratory and cardiac variables were recorded continuously.

During the second session, subjects were asked to perform a standard warm-up: 8 min at 150 W, 2 min at 260 W, a recovery period (i.e., 2 min), a 10-s sprint of progressively increasing intensity with the last 3 s performed at a maximal all-out intensity, 90 s of recovery and finally two brief all-out sprints (5 s in duration) interspersed with 90 s of recovery. After a further 10 minutes of passive recovery, subjects performed the cycling exercise (Tlim_supra_) at a constant power output (P_supra_Δ30%) for as long as possible until exhaustion. P_supra_Δ30% was defined as the supra-maximal intensity above MAP corresponding to an increment of 30% of the difference between P_max_ (estimated from torque-velocity test) and . Subjects were required to keep a constant pedalling rate (i.e., corresponding to *f*_opt_ minus 10%). No information relative to test duration was given to the subjects. The test continued until complete exhaustion: either until the cyclists voluntarily chose to stop the exercise or until they were no longer able to maintain their initial test cadence (± 3 rpm), which was considered as a failure to maintain the required task (i.e., the target power output at a constant cadence). Respiratory and cardiac responses were recorded continuously during the entire experimental session. Arterialised capillary blood samples (85 μL) were taken from a hyperemized ear-lobe just before the start of Tlim_supra_ (7 min after the end of the warm-up), at exhaustion, and 5 and 8 min during the passive recovery.

### Material and data collection/processing

All testing sessions took place in a well-ventilated laboratory at a temperature of 20–22°C and were conducted using an electronically-braked cycle ergometer (Excalibur Sport, Lode, Groningen, The Netherlands). Vertical and horizontal positions of the saddle, handlebar height, crank and stem lengths were set to match the most comfortable and usual position of the participants.

### Respiratory responses

Spirometric variables, [i.e. forced vital capacity (FVC), forced expiratory volume in 1 s (FEV_1_), Tiffeneau index (FEV/FVC), forced inspiratory volume (FIVC) forced inspiratory volume in 1 s (FIV_1_), forced expiratory flow at that point that is 25, 50 or 75% from FVC (FEV_25, 50 or 75_)] (Figure 2A) were measured with an ergospirometric device (Spirobank II, MIR, Roma, Italy) before and 3 min after the end of Tlim_supra_. The precision and reproducibility of the data (FEV_1_ and FVC) have been reported (Liistro et al. 2006). Before Tlim_supra_, a minimum of three satisfactory inspiratory and expiratory efforts were conducted with the highest measurement being defined as maximal. At the end of the Tlim_supra_, and due to time-constraints (recovery influence), only one satisfactory measurement was asked to the subjects in order to measure the exercise-induced changes in the respiratory function.

During both IT and Tlim_supra_, , , CO_2_ production , respiratory frequency (RF), V_T_ and end-tidal oxygen tension (P_ET_O_2_) were recorded breath by breath with a fixed gas exchange system (Quark CPET, Cosmed, Roma, Italy). Calibration of the gas analyser was performed according to the manufacturer’s instructions before each test for each subject. To avoid artefacts in recording signals, the finger was warmed with a vasodilator ointment 10–15 min before starting the measurement. The apparatus was automatically calibrated before each test. During the IT, breath-by-breath gas exchange values were smoothed (i.e., 3-s central moving average). In order to characterize the subjects, the highest  value in a 30-s period was considered as the . The criteria used for the determination of  were threefold: a plateau in  despite an increase in power output, a respiratory exchange ratio (RER) above 1.1, and a heart rate (HR) above 90% of the predicted maximal HR. For the purpose of comparing, over the same period of sampling, with the peak value of  measured during Tlim_supra_, the highest 5-s average was also determined. To determine  during Tlim_supra_ (and as previously reported (Hanon et al. 2010)), values were smoothed (i.e. 3-s central moving average) and then a 5-s average was applied in order to compare  and other ventilatory responses (V_T_, RF, ), with those of cardiac output (CO), stroke volume (SV) and changes in SaO_2_ at the same time.

For Tlim_supra_, the end  value  was defined as the average during the last 5-s period and the  decrease was considered as . The  decline was considered as a  decrease, when the magnitude of the phenomenon was larger than 5% of the peak value while the power of exercise continued to be above  (Billat et al. 2009). The same criterion was applied to the other cardio-respiratory variables.

The  of the respiratory muscles (VRMO_2_, expressed in mL.min^-1^), was calculated from the work of breathing (W_B_, kg.min^-1^) using the equation proposed by Coast et al. (Coast et al. 1993):


The ventilatory reserve (VR) was defined as  expressed as a percent of the estimated resting MVV (maximal voluntary ventilation):


(Johnson et al. 1996)*.*

### Cardiac responses

A bio-impedance method was used to determine SV, HR and CO (Physioflow, Manatec Type PF05L1, Strasbourg, France). The basis for this technique and its application, validity and reliability for exhaustive exercise testing have been described (Lepretre et al. 2004), and it has been demonstrated that thoracic hyperinflation does not alter CO (Charloux et al. 2000). For this experiment, SV, HR and CO values were averaged every five seconds.

### Blood metabolic responses

Prior to, 0 and 3 min post-IT, blood samples were collected and analysed for lactate concentration using a Lactate Pro analyser (Arkray, Japan). Prior to and post-Tlim_supra_ session, arterialised capillary blood samples (85 μL) were analysed to measure blood pH, [La], SaO_2_, PaO_2_ and CO_2_ (PaCO_2_) and bicarbonate concentration ([HCO_3_^-^]) with an i-STAT dry chemistry analyser (Abbott, Les Ulis, France).

### Statistical analysis

Data are reported as mean ± SD. Because subjects did not perform exactly the same exercise duration, data were expressed relative to the % of total duration (every 5% of Tlim_supra_ duration) for Figure 1 and for ANOVA. Changes in gas-exchange variables during Tlim_supra_ were evaluated by a one-way analysis of variance (ANOVA), with repeated-measures across each 5% interval, followed by multiple comparisons (Student-Newman-Keuls) to test the effect of time on the variables. The intra-class correlation (ICC) was calculated for pre-test spirometric data. Relationships between variables (ventilatory, cardio-dynamic, arterial oxygen saturation, metabolic parameters and ) at different times of the test and final Tlim_supra_ performance were analyzed by a Pearson’s correlation coefficient. In order to measure the strength of the relationship between the  decrease and a given variable, while controlling the effect of the other variables, Pearson partial correlations were also calculated. The level of significance was set at *P* < 0.05. Finally, aiming to compare the difference in main variables, between the subject who exhibited a > 5% decrease in  and the others, effect sizes (ES) were calculated using Cohen’s *d*. Effect sizes of 0.8 or greater, around 0.5 and 0.2 or less were considered as large, moderate, and small, respectively. The level of significance was set at *P* < 0.05.
